# External validation of the AntiEpileptic Drug Monitoring in PREgnancy (EMPiRE) model for predicting seizures in pregnant women with epilepsy

**DOI:** 10.1186/s12884-023-05822-z

**Published:** 2023-07-11

**Authors:** Yanru Du, Qi Xu, Jiahe Lin, Jiaoni Gong, Niange Xia, Zhenguo Zhu, Xinshi Wang, Rongyuan Zheng, Huiqin Xu

**Affiliations:** Department of Neurology, the First Affiliated Hospital of Wenzhou Medical University, Shangcai village, Ouhai District, Wenzhou, Zhejiang Province P.R. China

**Keywords:** Pregnant women with epilepsy, Predict seizures, The EMPiRE model, External validation

## Abstract

**Background:**

The AntiEpileptic Drug Monitoring in PREgnancy (EMPiRE) model is the only available tool for predicting seizures in pregnant women with epilepsy (WWE) using anti-seizure medications (ASMs); however, its predictive performance requires validation. This study aimed to evaluate the predictive ability of this model in pregnant Chinese WWE and its potential usefulness in clinical practice.

**Methods:**

Data of the EMPiRE model were derived from the EMPiRE study, a prospective multicenter cohort study that recruited women on ASM monotherapy (lamotrigine, carbamazepine, phenytoin or levetiracetam) or polytherapy (lamotrigine with either carbamazepine, phenytoin or levetiracetam). Based on the applicable population of the EMPiRE model, we evaluated 280 patients registered in the Wenzhou Epilepsy Follow-up Registry Database from January 1, 2010, to December 31, 2020. A total of 158 eligible patients were included in the validation cohort. We collected data on the baseline characteristics of patients, eight predictors of the EMPiRE model and outcome events. The outcome was the occurrence of tonic-clonic or non-tonic-clonic seizures at any time in pregnancy up to 6 weeks postpartum. We used the equation of the EMPiRE model to obtain the predicted probabilities of seizures. The predictive ability of the EMPiRE model was quantified by the C-statistic (scale 0–1, values > 0.5 show discrimination), GiViTI calibration test and decision curve analysis (DCA).

**Results:**

Of 158 eligible patients, 96 patients (60.8%, 96/158) experienced one or more seizures at any time between pregnancy and 6 weeks postpartum. The EMPiRE model showed good discrimination with a C-statistic of 0.76 (95% confidence interval [CI] 0.70–0.84). The GiViTI calibration belt showed that the predicted probabilities, which ranged from 16 to 96% (95% CI), were lower than the actual probabilities. DCA indicated that the highest net proportional benefit was obtained for predicted probability thresholds of 15–18% and 54–96%.

**Conclusions:**

The EMPiRE model could discriminate well between WWE with and without seizures during pregnancy and 6 weeks postpartum, but the risk of seizures may be underestimated. The limitations of the model for specific medication regimens may limit its real-world application. If the model is further improved, it will be incredibly valuable.

**Supplementary Information:**

The online version contains supplementary material available at 10.1186/s12884-023-05822-z.

## Background

Epilepsy is a common neurological disorder characterized by recurring seizures and requires long-term treatment with anti-seizure medications (ASMs) [1]. Statistics show that approximately 12.5 million women of childbearing age worldwide have epilepsy [2] and that 30–70% of pregnant women with epilepsy (WWE) use ASMs [3–5]. The management of epilepsy during pregnancy presents substantial challenges. Physicians must balance the maternal risk of seizures and the fetal teratogenicity of ASMs [1]. The reports that WWE face an increased risk of death from seizures during pregnancy. A 2004 study in the United Kingdom estimated 10 times higher maternal mortality in WWE than in those without epilepsy [6]. Moreover, tonic-clonic seizures can cause injury from drowning, motor vehicle accidents, and falls [7]. Seizures in pregnancy also have a negative impact on daily living. For example, the loss of a driver’s license following a seizure affects employment, relationships, and quality of life [8–10]. Furthermore, abrupt withdrawal of ASMs could provoke life-threatening seizures [1]. A questionnaire-based study showed that more than 50% of WWE decided to discontinue their ASMs during a future pregnancy, and this decision is associated with low levels of pregnancy-related knowledge [11]. Therefore, predicting the probability of seizures during pregnancy and identifying high-risk patients are essential for reducing epilepsy-related maternal deaths, improving patients’ quality of life, and promoting patient adherence to medication. However, to date, only one model has been available to predict the risk of seizures in pregnant WWE during pregnancy and up to 6 weeks postpartum.

In 2019, the AntiEpileptic Drug Monitoring in PREgnancy (EMPiRE) model was developed and validated to predict seizure risk at any time in pregnancy and until 6 weeks postpartum in WWE on ASMs [12]. The EMPiRE model used the datasets of 527 pregnant WWE treated with ASM monotherapy (lamotrigine, carbamazepine, phenytoin or levetiracetam) or polytherapy (lamotrigine with either carbamazepine, phenytoin or levetiracetam) recruited from 50 hospitals in the United Kingdom [13]. The development cohort comprised 399 women, and the validation cohort comprised 128 women. The researchers reported that the EMPiRE model performed well, with a C-statistic of 0.79 (95% confidence interval [CI], 0.75–0.84) and calibration slope of 1.26 (95% CI 0.98–1.54). The EMPiRE model discriminated well between WWE with and without seizures. On external validation, the model also showed good discrimination (C-statistic of 0.76, 95% CI 0.66–0.85) but with imprecise estimates (calibration slope 0.93, 95% CI 0.44–1.41) [13].

The EMPiRE model is currently the only available tool for predicting seizure risk in pregnant WWE. Predicting seizures based on women’s individual characteristics not only provides accurate risk information for individualized decision-making but also facilitates effective communication with physicians and patients. Therefore, if the EMPiRE model can be applied to the clinic, it will be very valuable. Prediction models must be validated in independent patient populations before being applied in routine clinical practice and external promotion, but the EMPiRE model has not been validated in China thus far. The aim of this study was to externally validate the predictive performance of the EMPiRE model in pregnant Chinese WWE at a comprehensive tertiary hospital and evaluate its clinical usefulness.

## Methods

### Study design and source of data

We selected eligible patients from the Epilepsy Long-term Follow-Up Registry Study (ELFURS) and formed a cohort to externally verify the performance of the EMPiRE model in three aspects: C-statistic, accuracy (GiViTI calibration test) and decision curve analysis (DCA). ELFURS was a prospective observational study conducted in 2003 at the epilepsy center of the First Affiliated Hospital of Wenzhou Medical University, a comprehensive tertiary hospital in China. ELFURS was introduced in a previous publication [14]. ELFURS was approved by the Ethics Committee of our hospital and registered on the World Health Organization Registry Network (registration number: ChiCTR-OCH-14,004,616). Patients enrolled in ELFURS were required to keep seizure diaries and videos with the help of family members and guardians and were followed up every 1 to 3 months at the epilepsy outpatient unit. We also simultaneously established the Wenzhou Epilepsy Follow-Up Registry Database (WEFURD) to record, save, and process the registration data. WEFURD was part of the ELFURS and was approved by the Ethics Committee of our hospital. WEFURD was managed by epilepsy specialists on our team. The WEFURD contains detailed data on patients diagnosed with epilepsy, including demographic information, clinical characteristics, epileptic seizures, and ASM prescriptions.

### Participants

In this study, based on the applicable population of the EMPiRE model, we searched the WEFURD for pregnant WWE between January 1, 2010, and December 31, 2020, and then evaluated them according to the following eligibility criteria. The inclusion criteria were (1) women diagnosed with epilepsy [15, 16]; (2) pregnant women on ASMs with a viable pregnancy; and (3) women who were receiving lamotrigine monotherapy/polytherapy (with carbamazepine, phenytoin or levetiracetam) or carbamazepine monotherapy or phenytoin monotherapy or levetiracetam monotherapy at the time pregnancy was confirmed, and types of their medications remained unchanged during pregnancy and up to 6 weeks after delivery, with or without adjustment of dosage. Notably, we monitored the concentrations of therapeutic medications in some WWE during pregnancy, and the medication dosages of some patients may be adjusted for several situations, e.g., when patients had unexpected seizure aggravation or when medication concentrations were significantly lower than prepregnancy levels. The exclusion criteria were (1) women age < 16 years; (2) information on all predictors that was not available; (3) taking none of ASMs in pregnancy; (4) use of other ASMs for monotherapy (excluding lamotrigine, carbamazepine, phenytoin and levetiracetam) in pregnancy; (5) women on non-lamotrigine polytherapy in pregnancy; (6) a history of alcohol or substance abuse/dependence in the last 2 years before pregnancy; and (7) participation in a clinical drug trial in pregnancy.

The study was approved by the Ethics Committee of our hospital (number 201,347), and written consent was obtained from all individual participants included in the study. For minors, informed consent was obtained from a parent and/or legal guardian. We reported the validation of the EMPiRE model with reference to the TRIPOD (Transparent Reporting of a multivariable prediction model for Individual Prognosis Or Diagnosis) recommendations [17] (see Additional file 1).

### Predictor variables and data collection

The EMPiRE model included eight predictors: age at first seizure, baseline seizure classification (tonic-clonic, non-tonic-clonic, unspecified), history of mental health disorder or learning difficulty, occurrence of tonic-clonic and non-tonic-clonic seizures in the 3 months before pregnancy, admission to the hospital for seizures in previous pregnancy, and baseline dose of lamotrigine and levetiracetam (mg/day) [12]. Data on all of the above predictor variables, baseline characteristics and the number and type of seizures of the patients during pregnancy and 6 weeks postpartum were collected from the WEFURD.

Prepregnancy baseline data included age, age at first seizure (excluding febrile seizures), seizure types, history of learning difficulty or mental illness, admission to hospital for seizures in previous pregnancy, type of seizures in the three months before pregnancy, and type and dosage of current ASMs. Seizure types were classified according to the most recent classification of the International League Against Epilepsy (ILAE) [15, 16, 18]. Tonic-clonic seizures include generalized onset tonic-clonic seizures, focal to bilateral tonic-clonic seizures and unknown onset tonic-clonic seizures. Non-tonic-clonic seizures include a focal aware seizure, a focal impaired awareness seizure, a nonmotor onset seizure, a myoclonic seizure and an absence seizure. Regarding the presence of developmental delay, the patients enrolled in ELFURS were first assessed by physicians, and then the Wechsler Adult Intelligence Scale test was administered to those with a possible developmental delay, which was conducted by psychologists in our hospital; patients with intelligence quotients of < 70 were considered to have a developmental delay. Further diagnosis of learning difficulties was diagnosed by psychologists and psychiatrists in our hospital in accordance with the Diagnostic and Statistical Manual of Mental Disorders (DSM) [19]. Mental health evaluations were conducted by mental health clinicians at outpatient visits and at follow-up visits. Patients who may have a mental illness, such as depression, puerperal psychosis, bipolar disorder and schizophrenia, were further evaluated and diagnosed by psychiatrists. The above relevant information was also recorded in the WEFURD.

In addition, we also collected data on medication adherence during pregnancy, as well as data on medication concentration monitoring, if available. For this study, we tried our best to contact patients face-to-face, by telephone or via letters to supplement missing data.

### Outcome event

The outcome event was the occurrence of tonic-clonic (convulsive) or non-tonic-clonic (nonconvulsive, e.g., myoclonic, absence) seizures over the whole period of pregnancy and 6 weeks postpartum [18].

### Calculation of the predicted probability of an outcome event

The patients’ predicted probabilities of seizures during pregnancy and until 6 weeks after delivery were calculated by means of the equation of the EMPiRE model [12]. The equation was as follows:

probability (seizure) = exp(Y)/(1 + exp(Y)).

where Y= − 1.39 + (–0.02*age at first seizure) + 0.61 [unspecified seizures] + 0.75 [non-tonic-clonic seizures] + (0.02*dose of levetiracetam/100) + (0.29*dose of lamotrigine/100) + 0.66 [non-tonic-clonic seizures in the 3 months before pregnancy] + 1.97 [tonic-clonic seizures in the 3 months before pregnancy] + 0.67 [learning difficulty or mental health disorder] + 0.17 [admitted to hospital for seizures during previous pregnancy].

All variables were coded as binary (1 when present and 0 when absent) except for age at first seizure (years), dose of lamotrigine (mg/day), and dose of levetiracetam (mg/day).

### Measurement of the EMPiRE model performance

The performance of the EMPiRE model was quantified by three aspects: discrimination (C-statistic), accuracy (GiViTI calibration test) and DCA. Discrimination is the ability to correctly distinguish individuals with different outcomes [20]. In this study, the C-statistic represents the ability of the model to discriminate patients with the outcome event (who experience seizures during pregnancy and 6 weeks postpartum) from patients without the outcome event (who had no seizures over the whole period of pregnancy and 6 weeks postpartum); a value of 1 indicates perfect discrimination, and a value of 0.5 indicates no predictive ability [17]. The C-statistic was quantified with the area under the receiver operating characteristic curve (AUC ROC) and its 95% CI. Models are considered to have good performance when the C-statistic exceeds 0.7 [21, 22]. We divided the study subjects into two groups, that is, patients with outcome events (i.e., patients with an observed risk of seizures) as group A and those without outcome events as group B. The predicted risk of seizures was calculated according to the formula of the EMPiRE model, as mentioned above. Accuracy refers to agreement between the predicted and observed risk of seizures for all groups of predicted probabilities. We used the GiViTI calibration test [23] to evaluate the difference between the predicted and observed probabilities. If its P value was not significant (e.g., greater than 0.05), the predictive model could be well calibrated on the considered sample, meaning that the predicted probabilities are almost equal to the observed proportions for all groups of predicted probabilities. Conversely, if the P value was significant, the calibration belt was considered. The calibration belt was a graphical tool to determine where the observed probabilities deviate from the expected probabilities. DCA was performed to measure the clinical usefulness (i.e., the ability to make better decisions with a model than without it) of the prediction models by quantifying the net benefits at different threshold probabilities [24]. DCA identified the appropriate range of threshold probability of the prediction model.

### Statistical analysis

Categorical data are presented as numbers (percentages), and normally distributed quantitative data are shown as the mean ± standard deviation (SD). We further analyzed the differences between Group A (the patients who had one or more seizures during pregnancy and 6 weeks postpartum) and Group B (the patients who had no seizures over the whole period of pregnancy and 6 weeks postpartum). The baseline characteristics of the two groups, including the eight predictors, were compared by univariate analysis using the two-sample t test, the chi-square test or Fisher’s exact test for heterogeneity, as appropriate. Specifically, the variables of age at first seizure, baseline dose of lamotrigine and levetiracetam were tested by the two-sample t test; the variables of baseline seizure classification, occurrence of tonic-clonic seizures in the 3 months before pregnancy, and occurrence of non-tonic-clonic seizures in the 3 months before pregnancy were tested by the chi-square test; and the variables of history of learning difficulty or mental illness and admission to hospital for seizures in previous pregnancy were tested by Fisher’s exact test. All tests were 2-sided, with statistical significance set at P < 0.05. Univariate analyses were performed using IBM SPSS Statistics 21.0 software (IBM Corporation, NY, USA). The C-statistic, GiViTI calibration test and DCA analyses were performed using R version 3.6.0 (R Project for Statistical Computing) with the pROC, ggplot2, givitiR, and rmda libraries (http://lib.stat.cmu.edu/R/CRAN/). A P value < 0.05 was considered statistically significant.

## Results

### Selection of eligible participants for external validation

**Fig. 1:**
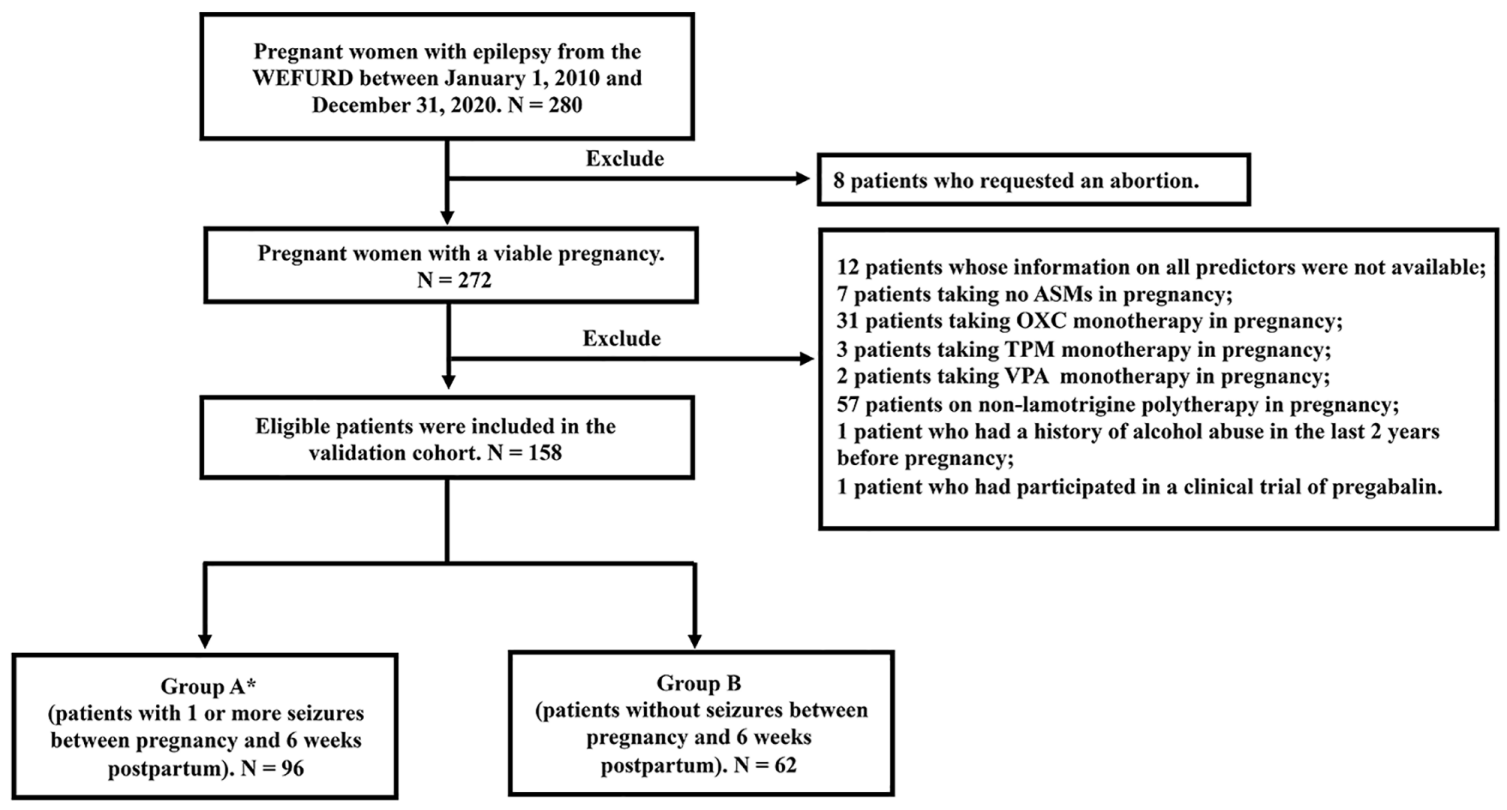
Flowchart of the selection of the validation sample based on the inclusion and exclusion criteria. Note: ASMs, anti-seizure medications; OXC, oxcarbazepine; TPM, topiramate; VPA, valproate; WEFURD, Wenzhou Epilepsy Follow-up Registry Database. *: 14 patients with the occurrence of only tonic-clonic seizures between pregnancy and 6 weeks postpartum. 64 patients with the occurrence of only non-tonic-clonic seizures (e.g., myoclonic, absence) between pregnancy and 6 weeks postpartum. 18 patients with the occurrence of both tonic-clonic seizures and non-tonic-clonic seizures between pregnancy and 6 weeks postpartum

Figure 1 shows the flowchart of the selection of the validation sample based on the inclusion and exclusion criteria. A total of 158 eligible patients were included in the validation cohort. Overall, there were 96 patients with the outcomes (Group A), i.e., experienced 1 or more seizures during pregnancy and 6 weeks postpartum, and patients with the occurrence of tonic-clonic seizures accounted for a third (32/96, 33.3%); there were 62 patients without the outcome (Group B), i.e., they had no seizures during pregnancy or by 6 weeks postpartum.

### Patient characteristics

Table 1 shows the baseline characteristics of 158 eligible patients and the results of univariate analysis for groups A and B. The average age at baseline of our validation cohort was 27.3 years (SD 4.4), and there was no significant difference between Group A and Group B (Table 1). The age at first seizure was different in the two groups (Group A, 15.9 ± 7.6 years; Group B, 18.3 ± 5.6 years, P = 0.02). The percentage of tonic-clonic seizures at baseline in our validation cohort was 85.4% (135/158), and the corresponding percentages were 83.3% (80/96) and 88.7% (55/62) in Groups A and B, respectively, with no statistically significant difference. However, the incidence of non-tonic-clonic seizures in the 3 months prior to pregnancy in the two groups significantly differed (Group A vs. Group B: 47/96, 49.0% vs. 4/62, 6.5%; P < 0.001). Lamotrigine was the most common ASM prescribed in both groups. A total of 38.6% (37/96) of Group A was treated with lamotrigine combined with levetiracetam, while more than half of the women in Group B took lamotrigine monotherapy (34/62, 54.8%).

**Table 1:** Baseline characteristics of 158 eligible patients and the univariate analysis in groups A and B

Characteristic	Validation cohort N = 158	Group A (patient with 1 or more seizures) N = 96	Group B (patient without seizure) N = 62	*p* value
Age at baseline (years)	27.3 ± 4.4	26.9 ± 4.3	27.9 ± 4.4	0.18
History of learning difficulty or mental illness^§^	4 (2.5%)	3 (3.1%)	1 (1.6%)	1.00^a^
Age at first seizure (years)	16.8 ± 6.9	15.9 ± 7.6	18.3 ± 5.6	0.02
≤ 10 years	30 (19.0%)	25 (26.0%)	5 (8.1%)	
11–20 years	91 (57.6%)	53 (55.2%)	38 (61.3%)	
21–30 years	33 (20.9%)	14 (14.6%)	19 (30.6%)	
31–40 years	4 (2.5%)	4 (4.2%)	0	
Admission to hospital for seizures in previous pregnancy	4 (2.5%)	4 (4.2%)	0	0.16^a^
Seizure classification at baseline				0.35
Tonic-clonic*	135 (85.4%)	80 (83.3%)	55 (88.7%)	
Non-tonic-clonic^#^	23 (14.6%)	16 (16.7%)	7 (11.3%)	
Tonic-clonic seizure* in the 3 months before pregnancy	8 (5.1%)	6 (6.3%)	2 (3.2%)	0.40
Non-tonic-clonic seizure^#^ in the 3 months before pregnancy	51 (32.3%)	47 (49.0%)	4 (6.5%)	< 0.001
Antiepileptic drug intake at baseline				
Carbamazepine	3 (2.0%)	2 (2.1%)	1 (1.6%)	1.00^a^
Levetiracetam	47 (29.7%)	24 (25.0%)	23 (37.1%)	0.11
Lamotrigine	65 (41.1%)	31 (32.3%)	34 (54.8%)	0.005
Lamotrigine and levetiracetam	41 (26.0%)	37 (38.6%)	4 (6.5%)	< 0.001
Lamotrigine and carbamazepine	1 (0.6%)	1 (1.0%)	0	1.00^a^
Lamotrigine and levetiracetam and carbamazepine	1 (0.6%)	1 (1.0%)	0	1.00^a^
Baseline dose of antiepileptic drugs (mg/day)				
Carbamazepine	680.0 ± 303.3	750 ± 300	400 ± NA	0.37
Lamotrigine	263.2 ± 163.5	276.1 ± 167.3	239.5 ± 155.6	0.27
Levetiracetam	1604.5 ± 1018.1	1783.1 ± 1042.4	1194.4 ± 841.6	0.01

Note: Values are expressed as a number (percentage, %) or mean ± standard deviation

NA, not applicable

^a^: Fisher’s exact test

^§^: One patient was diagnosed with learning disabilities, two patients were diagnosed with depression, and one patient was diagnosed with schizophrenia

*: Tonic-clonic seizures include generalized onset tonic-clonic seizure, focal to bilateral tonic-clonic seizure and unknown onset tonic-clonic seizure

^#^: Non-tonic-clonic seizures include a focal aware seizure, a focal impaired awareness seizure, a nonmotor onset seizure, a myoclonic seizure and absence seizure

Nineteen patients (19/158, 12.0%) had records of missing their medication or self-withdrawal medication during pregnancy. After compliance education, none of these 19 patients had medication omission or withdrawal records at subsequent follow-up. Of these 19 patients, 14 patients (14/96, 14.6%) were in Group A, and 5 patients (5/62, 8.1%) were in Group B, with a p value of 0.22 for the statistical result of the chi-square test. A total of 33 patients in our study were monitored for medication concentrations during pregnancy. Among them, 13 patients received lamotrigine monotherapy, with a mean concentration of 4.88 µg/ml; 4 patients received levetiracetam monotherapy, with a mean concentration of 18.90 µg/ml; and 16 patients received lamotrigine and levetiracetam combined therapy, with a mean concentration of lamotrigine of 3.81 µg/ml and levetiracetam of 21.74 µg/ml. The reference range for lamotrigine in our hospital is 2.5 µg/ml to 15.0 µg/ml. The reference range for levetiracetam in our hospital is 12.0 µg/ml to 46.0 µg/ml.

### Performance of the model in external validation

**Fig. 2:**
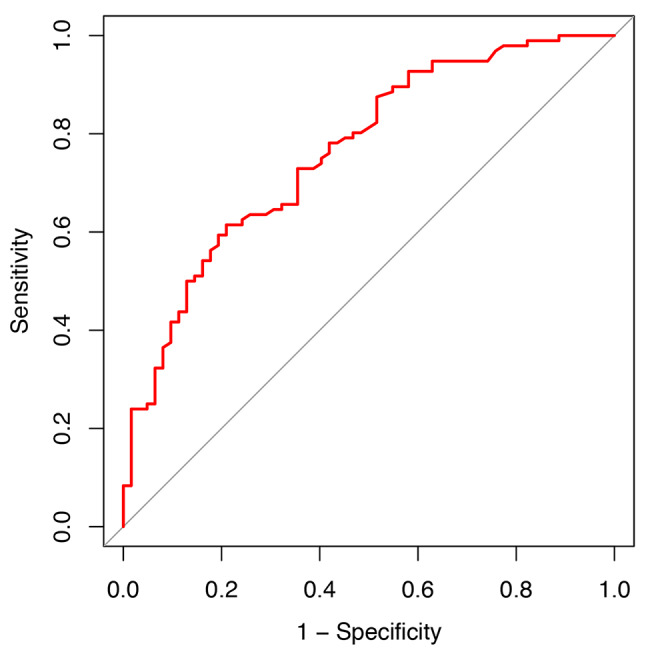
The receiver operating characteristic (ROC) curve of the EMPiRE model. Note: EMPiRE, AntiEpileptic Drug Monitoring in PREgnancy.

**Fig. 3:**
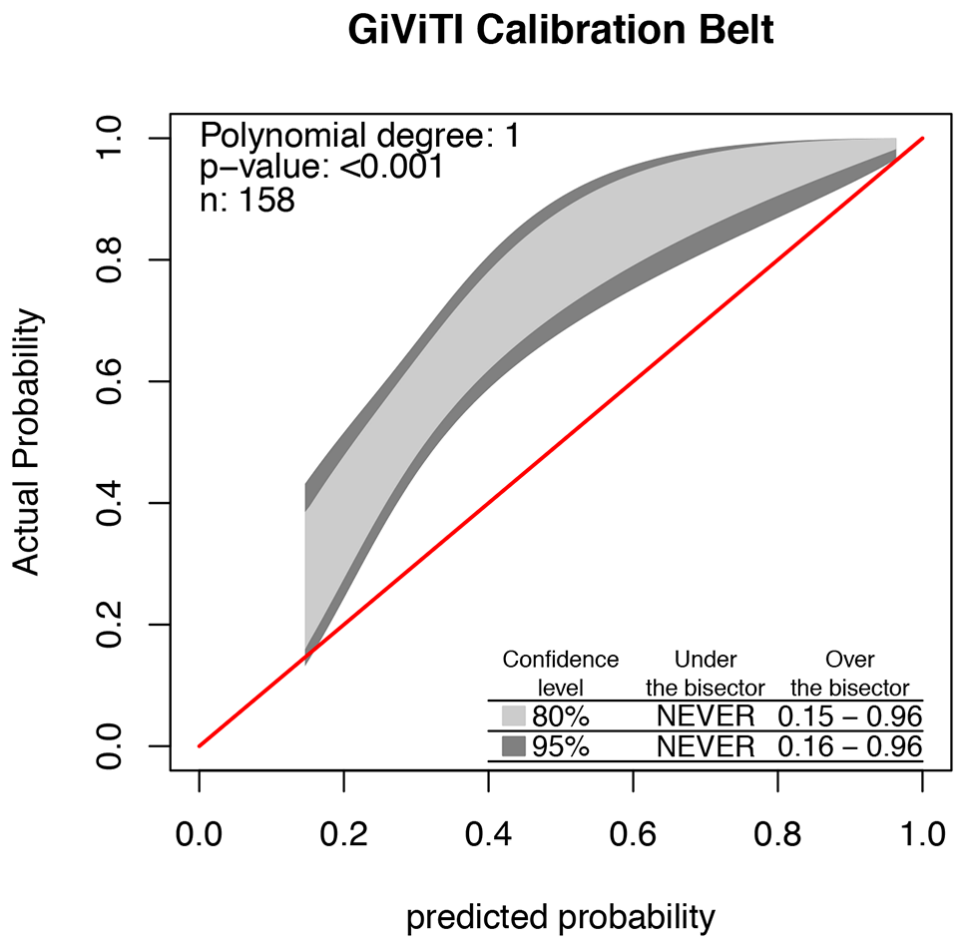
The GiViTI calibration belt of the EMPiRE model. Note: EMPiRE, AntiEpileptic Drug Monitoring in PREgnancy.

**Fig. 4:**
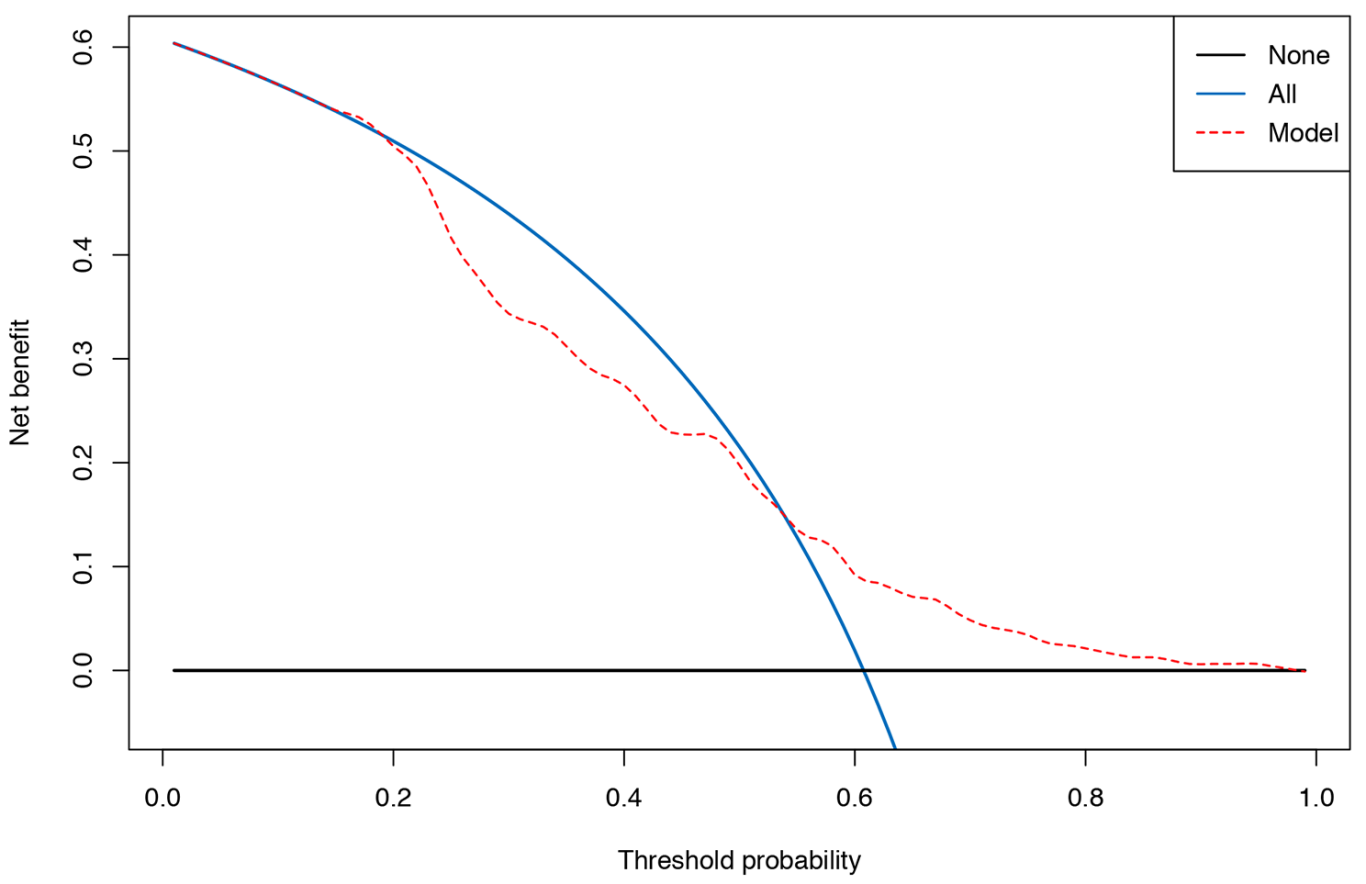
Decision curve analysis of the EMPiRE model. Note: Black line (treat none) = net benefit when we assume that no pregnant woman with epilepsy will have the outcome (seizure in pregnancy); blue line (treat all) = net benefit when we assume that all pregnant women with epilepsy will have the outcome; red line (the EMPiRE model) = net benefit when we manage pregnant women with epilepsy according to the predicted risk of the outcome (seizure in pregnancy) estimated by the EMPiRE model. EMPiRE, AntiEpileptic Drug Monitoring in PREgnancy.

Overall, 60.8% (96/158) of women in our validation cohort experienced 1 or more seizures between pregnancy and 6 weeks after delivery. The ROC curve of the EMPiRE model is plotted in Fig. 2. The C-statistic for the model was 0.76 (95% CI 0.70–0.84), and the model could discriminate well between WWE with and without seizures. However, the EMPiRE model was not accurate (GiViTI calibration test, P < 0.001). The GiViTI calibration belt in Fig. 3 shows the accuracy of the predicted and observed risks. According to the GiViTI calibration belt, the EMPiRE model may underestimate the risk of seizures in our validation population when the predicted probabilities are 16–96% (95% CI) and 15–96% (80% CI). For example, if the predicted probability (abscissa) is 40%, the actual probability (ordinate) is approximately 60–80%. In the DCA (Fig. 4), the curve for the EMPiRE model showed a positive net benefit for predicted probability thresholds of 15–18% and 54–96% compared to the strategies of assuming that all or none of the patients had seizures during pregnancy or by 6 weeks after delivery.

## Discussion

Seizures during pregnancy in WWE, especially tonic-clonic seizures, increase the risks of both mothers and fetuses [6, 7]. Thus, identifying WWE at high risk of seizures and further estimating the probability of the occurrence of seizures based on a woman’s individual characteristics are significantly helpful for the management of WWE during pregnancy. The EMPiRE model is currently the only model available for predicting the risk of seizures in pregnant WWE using ASMs, but it has not been validated in the Chinese population. In the present study, we collected data on 158 patients from a registry database at a comprehensive tertiary hospital in China, including eight predictors in the EMPiRE model and other characteristics, to externally evaluate the performance of the EMPiRE model. Our results indicate that the EMPiRE model showed good capacity for distinguishing between WWE with and without seizures (C-statistic, 0.76). However, in our validation cohort, the model may have underestimated the probability of seizures when the predicted probabilities ranged from 16 to 96% (95% CI), which may lead to misleading decisions in providing care for high-risk pregnancy conditions. DCA only shows a net benefit when the predicted risk probabilities are between 15 and 18% and 54–96%. In addition, the limitations of specific medicine therapies may limit the clinical application of this model. Outcomes predicted by the EMPiRE model included tonic-clonic seizures and non-tonic-clonic seizures. Considering the high risk of tonic-clonic seizures to the mother and fetus, we believe that it is more valuable to further optimize the model to predict the possibility of tonic-clonic seizures during pregnancy and by 6 weeks postpartum alone. We propose that the EMPiRE model may be optimized or redeveloped from the following points: (1) the outcome events only include tonic-clonic seizures during pregnancy and up to 6 weeks postpartum, and (2) history of tonic-clonic seizures in the 9 months before pregnancy, history of tonic-clonic seizures in the 6 months before pregnancy, and refractory epilepsy should also be considered as candidate predictors. Due to the small sample size of our study and few tonic-clonic outcome events in our study, it is difficult for us to improve the EMPiRE model, which we regret. We look forward to achieving this goal in the future through multicenter collaboration and the accumulation of more patients.

Our research shows that the EMPiRE model performs well in distinguishing between WWE with and without seizures. The C-statistics of our external validation cohort and that of the EMPiRE Model development cohort [12] were above 0.7. The P value of the GiViTI calibration test in our study was significant, p value < 0.001, so we further performed the GiViTI calibration belt. We found that this model may underestimate the probability of seizures. In the validation cohort of Shakila Thangaratinam et al., they also reported that the EMPiRE model may provide imprecise estimates (calibration slope 0.93, 95% CI 0.44–1.41) [12]. The features of different data sources may explain the differences in results. If a patient’s risk of seizures during pregnancy is underestimated, it may lead to poor decisions regarding treatment options and providing care. Reportedly, WWE may have an increased risk of premature contractions [25], and they have a higher risk of preterm labor and cesarean Sect. [26]. Seizures during pregnancy can also lead to low-birth-weight infants, preterm delivery, and small-for-gestational-age infants [27]. The management of pregnant WWE requires a multidisciplinary team of neurologists, obstetricians, midwives, and caregivers. It is extremely important to accurately identify high-risk groups to provide professional care and to develop preventive strategies to mitigate the effect of maternal epilepsy on pregnancy and perinatal outcomes.

The EMPiRE model was developed using data from the EMPiRE study, which is a double-blind randomized trial on the effectiveness and acceptability of monitoring strategies [13]. The EMPiRE study recruited 527 pregnant WWE on ASM monotherapy (lamotrigine, carbamazepine, phenytoin or levetiracetam) or polytherapy (lamotrigine with either carbamazepine, phenytoin or levetiracetam) from 50 hospitals in the United Kingdom [13]. The model development cohort comprised 399 women whose ASMs were adjusted based on clinical features only; the validation cohort comprised 128 women whose drug dose adjustments were informed by serum drug levels [12, 13]. The randomized trial study has high-quality data, but treatment medicines are fixed. In clinical practice, there are many more ASM regimens than those described above that are used during pregnancy; for example, many patients take oxcarbazepine monotherapy or combined with levetiracetam. In our study, our data came from a real-world registry study, which is more consistent with the actual clinical situation. We excluded 31 cases involving oxcarbazepine monotherapy and 57 cases involving non-lamotrigine polytherapy during pregnancy according to the applicable conditions of the model. Due to the limitation of specified therapy regimens, the validation sample size of this study was reduced, and the limitation of therapy regimens also limited the application and promotion of the model in clinical practice. Admittedly, another reason for the small sample size is that our patients came from a single center.

We consider that the different characteristics of the study population may also be related to the underestimation of the probability of seizures during pregnancy and by 6 weeks postpartum by the EMPiRE model in our validation cohort. Compared with the development data and validation data of Shakila Thangaratinam et al. [12], the distribution of the two predictor variables in our validation data is significantly different. One variable was seizure classification at baseline, and the other was tonic-clonic seizure in the 3 months before pregnancy. In the equation of the EMPiRE model, the coefficient of non-tonic-clonic seizures was 0.75. The proportion of non-tonic-clonic seizures at baseline in our study cohort was 14.6%, much lower than the 58% and 61% of the model development and validation cohorts, respectively [12]. Another important difference from the model development and validation cohorts is that the percentage of tonic-clonic seizures in the 3 months before pregnancy in our validation population was also low (our: 8/158, 5.1% vs. the development cohort: 52/399, 13.0% vs. the validation cohort 12/128, 9%). In the equation, the coefficient of tonic-clonic seizures in the 3 months before pregnancy was 1.97, which was the highest coefficient among all of the predictors. The differences mentioned above may be the reason for inaccurate estimates by the model.

The EMPiRE model also needs some improvement in the predictor variables. Guidelines recommend that WWE should be counseled that seizure freedom for at least 9 months prior to pregnancy is probably associated with a high rate (84–92%) of remaining seizure free during pregnancy (Level B) [25–33]. Data from the Australian Register of Antiepileptic Drugs in Pregnancy indicate that the risk of seizures during pregnancy was 50–70% less if the prepregnancy year was seizure free [34]. Therefore, it has been considered that a different predictor, such as a history of seizures in the 9 months before pregnancy rather than a 3-month history, may improve the performance of the EMPiRE model.

## Conclusions

The EMPiRE model contains eight clinically accessible variables and is the only clinical prognostic model that can be used to calculate the individualized risk of seizures in pregnant WWE on ASMs. Our study is the first study worldwide to evaluate the performance of the EMPiRE model in a Chinese population. Our results suggest that this model may need to be further refined. It would be far more valuable if the EMPiRE model could be optimized to predict only the probability of tonic-clonic seizures during pregnancy and be applied to all WWE. Our study has some limitations. First, all subjects of this study came from a single-center study at the specialized epilepsy clinic of a tertiary hospital, which might have caused selection bias. Second, the sample size of this study was not large enough. There are multiple pregnancy registries worldwide. We look forward to collaborating and further improving the EMPiRE model.

## Electronic supplementary material

Below is the link to the electronic supplementary material.

**Additional file 1** TRIPOD Checklist. Checklist items for prediction model development and validation. Note: TRIPOD, Transparent Reporting of a multivariable prediction model for Individual Prognosis Or Diagnosis.

## Data Availability

The datasets used and/or analyzed during the current study are available from the. corresponding author on reasonable request.

## Abbreviations

ASMs: anti-seizure medications
AUC ROC: area under the receiver operating characteristic curve
CI: confidence interval
DCA: decision curve analysis
ELFURS: Epilepsy Long-term Follow-Up Registry Study
EMPiRE: AntiEpileptic Drug Monitoring in PREgnancy
ILAE: International League Against Epilepsy
OXC: oxcarbazepine
SD: standard deviation
TPM: topiramate
TRIPOD: Transparent Reporting of a multivariable prediction model for Individual Prognosis Or Diagnosis
VPA: valproate
WEFURD: Wenzhou Epilepsy Follow-up Registry Database
WWE: women with epilepsy
